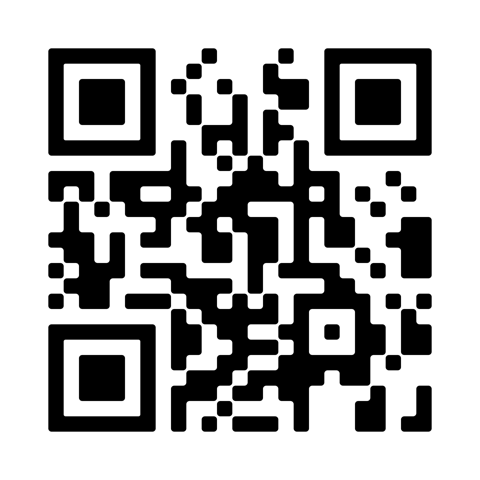# Laparoscopic management of a full-thickness uterine niche with subsequent pregnancy outcome

**DOI:** 10.52054/FVVO.13.4.038

**Published:** 2021-12-30

**Authors:** D.Z. Kasapoglu, L.Y.O. Tang, R.A. Kadir, F Shakir

**Affiliations:** Royal Free Hospital NHS Foundation Trust, Department of Obstetrics and Gynaecology

**Keywords:** Uterine niche, laparoscopy, repair, isthmocele

## Abstract

**Background:**

Uterine niche is the consequence of impaired healing of the myometrium following a lower segment transverse caesarean section (CS). Although there is conflicting evidence on the management of these cases, laparoscopic repair is a commonly used surgical treatment modality.

**Objectives:**

To demonstrate the management and laparoscopic repair of the niche with subsequent pregnancy outcome.

**Materials and Methods:**

We report a case of a 33-year-old patient who had a significant haematoma in the niche. The haematoma resolved after conservative management however, she remained symptomatic. Therefore, she had a laparoscopic repair. The narrated surgical video article demonstrates the dissection of the uterovesical fold overlying the niche, followed by the excision of the scar tissue and its repair with laparoscopic suturing. Ultrasound and magnetic resonance imaging images of the uterus demonstrating the haematoma at the caesarean section site, the niche after resolution of the haematoma and post-repair imaging are also provided.

**Main outcome measures:**

Repair of the niche, symptomatic relief of abnormal uterine bleeding, spontaneous conception and live birth. Ultrasonographic images also demonstrate uterine wall continuity post laparoscopic repair.

**Results:**

The patient recovered uneventfully. Full-thickness of myometrium was demonstrated with post-operative imaging and confirmed at the subsequent caesarean section. Gynaecological symptoms resolved following the repair. The patient conceived spontaneously after surgery and delivered at term by caesarean section without any complications.

**Conclusion:**

Laparoscopic management of the niche should be considered where there is a complete myometrial defect or significant thinning of the myometrium, especially in symptomatic women who desire future pregnancy.

## Learning objective

The decision-making process relating to the indications for performing a surgical repair of uterine niche ; planning with ultrasonographic and magnetic resonance imaging before surgery; techniques in laparoscopy including dissection and identification of the niche, resection of the fibrotic edge of the niche and suturing technique to close the defect.

## Introduction

Uterine niche, also known as isthmocele or caesarean scar defect, is the consequence of impaired healing of the myometrium following a low transverse caesarean section (CS) (Bij de Vaate et al., 2014). First described in 1995 as an unusual phenomenon, but with the increasing CS rates globally it is now a common clinical entity with a high prevalence of between 56%-84% (Morris 1995; Bij de Vaate et al., 2014; Betrán et al., 2016). Several hypotheses on the aetiology of niche development have been made but the reason for impaired tissue healing remains unknown (Vervoort et al., 2015). High body mass index (BMI), gestational diabetes, multiple previous CS scars and post-operative infection are risks factors for niche development in the lower segment (Antila-Långsjö et al., 2018).

The European Niche task force defined the niche as an indentation of at least 2mm at the previous CS scar (Naji et al., 2012; Jordans et al., 2019). Gel infusion sonography (GIS) is the gold standard diagnostic method and conventional transvaginal ultrasound has acceptable rates of diagnosis (Bij de Vaate et al., 2011; Roberge et al., 2012).

While women with niches mostly remain asymptomatic, a minority report irregular bleeding, continuous brown discharge, secondary infertility and abdominal pain (Van der Voet et al., 2014). The duration and severity of the symptoms described above have also been linked with the size of the niche (Tower and Frishman, 2013; Bij de Vaate et al., 2014; Schepker et al., 2015).

While the overall expert consensus is expectant management for asymptomatic niche s detected incidentally on pelvic imaging, laparoscopic repair is a commonly used surgical treatment modality that will be discussed further in this article (Nezhat et al., 2017; Brook et al., 2020).

## Patients and methods

A 33-year-old woman presented with abdominal pain after her 4^th^ delivery. She had a vaginal delivery, followed by an elective CS and two subsequent vaginal births. A significant haematoma was found within the CS scar on transvaginal ultrasound and magnetic resonance imaging (MRI). Initially, the haematoma was treated conservatively. However, she continued to have post-menstrual bloody vaginal discharge and intermenstrual bleeding which did not respond to tranexamic acid and combined contraceptive pills. A repeat MRI demonstrated resolution of the haematoma, yet a full-thickness myometrial defect remained.

## Intervention

*Step 1.* Identification of the niche was performed by reflecting the uterovesical peritoneum. The endocervical canal was demarcated using a size 8 Hegar dilator. The uterovesical dissection is continued to reveal the cervix distally to the niche thereby allowing adequate uterine and cervical tissue for reconstruction.

*Step 2.* The fibrous scar tissue surrounding the defect is excised until healthy myometrial tissue is clearly seen.

*Step 3.* Reconstruction of myometrium and endocervix was performed by using single-layer interrupted Vicryl-1 sutures. The sutures were then tied using a slip knotting technique to ensure that the tensioned edges were approximated appropriately.

*Step 4.* Hyalo-barrier adhesion gel was applied to the reconstructed area in an effort to reduce the risk of adhesion formation.

## Results

Recovery was uneventful following the surgery. A transvaginal ultrasound demonstrated myometrial continuity with a thickness of 10.3mm, 8 weeks following the operation. Symptoms of postmenstrual discharge and intermenstrual bleeding resolved completely. The patient conceived spontaneously within a year following the laparoscopic repair and myometrial thickness was 2.8mm at the anomaly scan. Due to the history of full-thickness uterine wall surgery, she was delivered at 38 weeks by elective low segment CS. During the CS, the lower segment appeared intact with no evidence of a defect or thinning of the myometrium. The success of surgical repair is evident by the cessation of symptoms, spontaneous conception, ultrasonographic measurements of the lower segment antenatally, macroscopic appearance at the time of subsequent caesarean delivery, and live birth at term (38 weeks’ gestation).

## Discussion

In the literature, the most common symptoms in women with niche are prolonged bleeding and vaginal spotting after menstruation (Bij de Vaate et al., 2014; Van der Voet et al., 2014). In our case, the patient presented with abdominal pain and abnormal vaginal bleeding. It is possible that the patient’s uterine scar was dehisced during the most recent vaginal delivery as she was completely asymptomatic following her first vaginal delivery after CS. This is an unusual presentation of the niche , highlighting that clinicians should be aware of the possibility of scar dehiscence after vaginal delivery when there is a history of previous uterine surgery.

Management of the niche should be tailored based on symptoms, residual myometrial thickness (RMT) and expectations on future fertility (Nezhat et al., 2017). Our patient continued to have vaginal bleeding despite hormonal treatment and she also desired future pregnancy. Therefore, laparoscopic management was considered an appropriate approach. Recent evidence shows a significant improvement in gynaecological symptoms following minimally invasive surgery (Van der Voet et al., 2014). In a large systematic review, the percentage of women reporting symptomatic relief was found to be 100% following laparoscopic repair and 87% after hysteroscopic management (Van der Voet et al., 2014). The hysteroscopic approach allows for the free drainage of menstrual blood by removing the distal ridge of the defect but this resection has also been shown to enlarge the size of the niche . In addition, patients with RMT less than 3mm were not eligible for hysteroscopic management due to the risks of perforation of the uterus, bladder and cervical injury (Vervoort et al., 2015). This method is also not suitable for women desiring pregnancy. Laparoscopic repair is a safe and effective option if RMT is less than 3mm where good fertility outcomes have been reported (Vervoort et al., 2018).

Current literature has described various laparoscopic techniques and approaches for resecting the niche . Primarily, the challenges include the identification of the niche at the time of surgery. Usually, this is clearly seen when blood and mucus from the niche are visualised following the reflection of the uterovesical peritoneum. Hegar dilators can be inserted into the intrauterine space to aid the visualisation of the area of defect that requires resection. This proves to be useful when ultrasonic devices are employed to excise the niche and during suturing to avoid an inadvertent puncture of any plastic manipulators. Laparoscopic instruments used for niche resection commonly include laparoscopic scissors and ultrasonic devices. In this case, we have used the Ethicon Harmonic ACE +7 Device (Ethicon Inc., Cincinnati, OH, USA) to excise the fibrous tissue around the niche . Single-layer continuous intra-corporeal interrupted suturing was completed to lower the risk of tissue necrosis. It is important to ensure that the inclusion of tissue is full-thickness (both proximally and distally to the defect) and the metal dilator aids the placement of the needle. Furthermore, by placing all the sutures before they are tied, the inclusion of full-thickness myometrium at the opposing edges is also guaranteed. Three interrupted sutures, with the placement of the middle suture prior to the distal sutures, allows for a good approximation.

## Conclusions

This video demonstrates the technique of laparoscopic niche repair including the steps of identification of the niche at the time of surgery with the insertion of a Hegar dilator via the endocervical canal, resection of the affected area using the Harmonic device and laparoscopic repair with single-layer interrupted suturing with coated Vicryl-1. The repair of the niche was successful as demonstrated by ultrasonographic measurements of uterine wall thickness, symptomatic relief and good pregnancy outcome subsequently.

This was an interesting case where the symptoms of the niche presented after the patients second vaginal birth following primary CS. This case reminds clinicians to be aware of the possibility of scar dehiscence after vaginal delivery in the presence of any previous uterine surgery.

## Video scan (read QR)

https://vimeo.com/590935926/7d40371b1c

**Figure:**